# Characterizing the in-out asymmetry in visual crowding

**DOI:** 10.1167/jov.21.11.10

**Published:** 2021-10-20

**Authors:** Ramakrishna Chakravarthi, Jirko Rubruck, Nikki Kipling, Alasdair D. F. Clarke

**Affiliations:** 1 School of Psychology, University of Aberdeen, Aberdeen, UK; 2 Department of Psychology, University of Essex, Essex, UK

**Keywords:** crowding, masking, inner-outer asymmetry (IOA), attention, location uncertainty

## Abstract

An object's processing is impaired by the presence of nearby clutter. Several distinct mechanisms, such as masking and visual crowding, are thought to contribute to such flanker-induced interference. It is therefore important to determine which mechanism is operational in any given situation. Previous studies have proposed that the in-out asymmetry (IOA), where a peripheral flanker interferes with the target more than a foveal flanker, is diagnostic of crowding. However, several studies have documented inconsistencies in the occurrence of this asymmetry, particularly at locations beyond the horizontal meridian, casting doubt on its ability to delineate crowding. In this study, to determine if IOA is diagnostic of crowding, we extensively charted its properties. We asked a relatively large set of participants (*n* = 38) to identify a briefly presented peripheral letter flanked by a single inward or outward letter at one of four locations. We also manipulated target location uncertainty and attentional allocation by blocking, randomizing or pre-cueing the target location. Using multilevel Bayesian regression analysis, we found robust IOA at all locations, although its strength was modulated by target location, location uncertainty, and attentional allocation. Our findings suggest that IOA can be an excellent marker of crowding, to the extent that it is not observed in other flanker-interference mechanisms, such as masking.

## Introduction

Objects in clutter are hard to identify (Bouma, 1970; Levi, 2008). This phenomenon, known as visual crowding (Stuart & Burian, 1962), has been extensively studied over the past several decades and has provided valuable insights into several cognitive processes, including object recognition (Levi, 2008; Whitney & Levi, 2011), reading (Martelli, Di Filippo, Spinelli, & Zoccolotti, 2009; Pelli, Tillman, Freeman, Su, Berger, & Majaj, 2007), and awareness (Atas, Faivre, Timmermans, Cleeremans, & Kouider, 2014; Kouider, Berthet, & Faivre, 2011). However, deleterious interactions between an object and its flankers can also occur due to other spatial processes, such as surround suppression and contrast masking (Levi, Hariharan, & Klein, 2002; Pelli, Palomares, & Majaj, 2004; Petrov, Popple, & McKee, 2007). It has been suggested that crowding and contrast masking mechanisms can be distinguished by a set of properties: masking impairs detection, whereas crowding only affects identification; masking scales with the size of the objects, whereas crowding does not; masking is independent of eccentricity, whereas crowding is intimately dependent on it (Levi et al., 2002; Pelli et al., 2004).

Hence, the diagnostic test to determine if a particular target has suffered from crowding and not from masking was to check if the spatial extent of the target-flanker interaction scaled with eccentricity but not with stimulus size. If it did, then it was crowding. However, this set of diagnostic criteria was challenged by Petrov et al. (2007), who argued that surround suppression also had the same set of features. They proposed that the unique property differentiating crowding and surround suppression was that crowding displayed a specific sort of asymmetry known as in-out asymmetry (IOA) that surround suppression did not. IOA is the finding that the flanker farther away from the fovea (the “outward” flanker) relative to the target interferes with target identification more than the flanker that is closer to the fovea (the “inward” flanker). This appears counter-intuitive since the inward flanker should be more perceptible, because of better acuity for objects closer to the fovea, and hence should lead to more interference. However, a recent study has argued that an immediately adjacent outward flanker dominates perception under crowded conditions (Shechter & Yashar, 2021). The increased interference from the outward flanker is often explained as a consequence of cortical magnification, due to which neurons that respond to the outward flanker are closer to the neurons responding to the target than are the neurons responding to the inward flanker (Motter & Simoni, 2007; Pelli, 2008), and hence cause more interference. Others have argued that the IOA can be attributed to the fact that receptive field sizes (within which interference occurs) increase with eccentricity (Dayan & Solomon, 2010) or that a higher weight is assigned to the immediately outward flanker when sampling from available features (Shechter & Yashar, 2021).

IOA has been noticed and reported in a variety of settings right from the early days of crowding research (Banks, Bachrach, & Larson, 1977; Bex, Dakin, & Simmers., 2003; Estes, Allmeyer, & Reder, 1976; Krumhansl, 1977; Mackworth, 1965; see Strasburger, 2020 for a thorough historical exegesis of the phenomenon). Consequently, Petrov et al. (2007) proposed that a demonstration of IOA should be considered diagnostic of crowding.

However, IOA is not observed in all situations. For example, Petrov and Meleshkevich (2011b) found that IOA was present only along the horizontal meridian and not at other locations. In fact, most previous studies where IOA has been demonstrated also seem to have documented it along the horizontal meridian (Petrov et al., 2007; Petrov & Meleshkevich, 2011a; Shechter & Yashar, 2021; Strasburger & Malania, 2013), whereas only a few studies have tested it at locations beyond the horizontal meridian (Bex et al., 2003; Petrov & Meleshkevich, 2011b). Even among the latter, one (Bex et al., 2003) did not actually test IOA at multiple locations, but instead tested stimuli that moved around a circular path around fixation (and therefore can be considered to be not restricted to the horizontal meridian). Hence, there is not much evidence for IOA at locations beyond the horizontal meridian. Further, Petrov and Meleshkevich (2011b) showed that IOA was stronger if participants had to monitor two spatial locations than if the target was consistently presented at the same location. They argued that this effect could be ascribed to attentional deployment. That is, they contended that strong IOA is observed if (endogenous) attention is deployed to two locations but is weaker or absent if it is allocated to a single location, which is the case when the target is predictably presented at the same location. These findings would undermine the utility of IOA as a diagnostic indicator, since crowding is observed even if the target is always presented at the same location and also at locations other than the horizontal meridian. That is, if IOA cannot be observed in these circumstances, then it cannot serve as a diagnostic tool.

Further evidence for the role of attention in IOA was provided when, in a different study, Petrov and Meleshkevich (2011a) found that focusing attention to be at the target location (while the target could be at one of two locations) increased IOA, whereas diffusing attention by randomly jittering the target location over a small region decreased the strength of IOA. Additionally, when participants had to attend a central location, IOA was eliminated or even reversed in some participants. This dependency (and indeed reversal) of IOA on the distribution of attention is another reason for a careful assessment of IOA as a diagnostic tool of crowding.

As discussed by Strasburger (2020), IOA appears to be reversed under some circumstances. When characters (letters or numerals) were used as stimuli, participants reported the inward flanker more often than the outward flanker in error trials (Chastain, 1982; Strasburger & Malania, 2013). These target-inward flanker confusions also scaled with eccentricity (Strasburger & Malania, 2013). These findings indicate that, when measured in a specific way, the effect of the inward flanker on the target appears to be stronger than that of the outward flanker. This seems to be the case when location uncertainty leads to whole objects being swapped among the target and its flankers. On the other hand, a recent study that used oriented Gabors combined with a continuous report measure found that the outward flanker is misreported far more often than the inward one when confusion does occur (Shechter & Yashar, 2021).

There are additional potential issues with the stimulus setup and participants generally used in these paradigms. Generally, these studies have been conducted with a few, experienced participants (which is often a good thing). Crucially, considerable interobserver variability has been documented (Greenwood, Szinte, Sayim, & Cavanagh, 2017; Petrov & Meleshkevich, 2011b). If a property is to be considered diagnostic, it should be evident consistently in most, if not all, observers and not just the experienced. Incidentally, Petrov and Meleshkevich (2011a, 2011b) used coarsely discriminable stimuli (identifying Gabor orientations differing by 90 degrees), which itself might result in little to no crowding (Pelli et al., 2004). This might have underestimated the strength and prevalence of IOA. Importantly, it could have concealed the existence of IOA in locations other than the horizontal meridian and for targets in predictable locations. Hence, stimulus choice might also be important in determining the robustness of IOA.

In the light of these inconsistent findings, can IOA be considered diagnostic of crowding? This study,^1^ therefore, addresses a few related issues: (1) Can IOA be observed in (a relatively larger sample of) naïve observers? If so, what is its prevalence within this sample and how reliably can it be detected? (2) What is the strength of IOA and how variable is it across individuals? (3) Is IOA restricted to the horizontal meridian, as claimed by Petrov and Meleshkevich (2011b)? If so, it cannot be considered diagnostic of crowding. (4) It was suggested that IOA depends on attentional deployment; this study will also test if this is the case by directly manipulating attentional deployment using pre-cues in a typical crowding paradigm. Finally, we will use letters as stimuli, which require fine discrimination and have been widely documented to be susceptible to crowding.

Importantly, we do not intend to question whether the extensively documented IOA exists or not, and, as such, there is little value in carrying out standard null hypothesis testing. Instead, the aim of our study is to measure the size of the asymmetry, and how it varies across participants over a range of locations and manipulations. Therefore, to characterize IOA, we use Bayesian multilevel models, which allow us to estimate the relevant parameters and the associated uncertainty.

## Methods

### Participants

Forty undergraduate students were recruited at two separate sites (20 at the University of Aberdeen and 20 at the University of Essex). All participants provided written informed consent and received monetary compensation. The study received prior approval from the respective local ethics boards and was conducted in accordance with the Declaration of Helsinki. All participants self-reported normal or corrected-to-normal vision. Two participants (from the University of Essex) did not complete all four sessions and hence were not included in our data analysis (final *n* = 38). Initial reports (Petrov et al., 2007) suggested that crowding threshold elevation was four times higher for the outward flankers compared to the inward flankers, which is a large effect. Indeed, IOA was observable in each of the four participants they tested. Later studies, when the effect was replicated, suggested slightly lower in-out ratios but still substantial enough to be observed in a few participants. Hence, a sample of 38 naïve participants, which is roughly three times larger than the largest sample tested previously,^2^ should be sufficient to detect it, if it exists, and to characterize its nature and prevalence.

### Materials and stimuli

**Figure 1.**
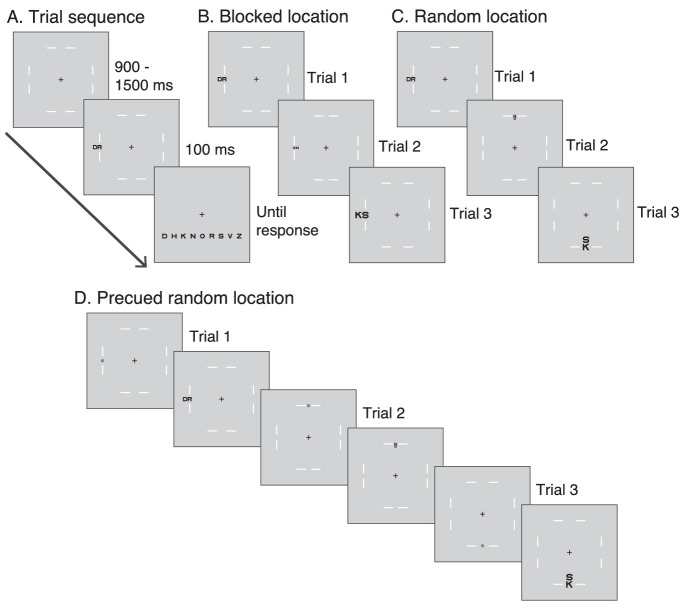
**Trial sequence and stimulus conditions.** Panel (**A**) illustrates the temporal sequence of a typical trial. After a fixation period between 900 and 1500 ms, the target and its flanker were presented for 100 ms. In this example, a single “outward” flanker was presented along with the target. However, in other trials, the flanker might be presented on the “inward” side or not presented (see panels **C** and **D**). The size of the letters (and hence spacing, which is set as 1.1*size) was controlled by the QUEST algorithm to determine flanked and unflanked acuity. All possible letter identities were then presented for the participant to make a choice. (**B**) Trials in the “blocked location” condition: targets were always presented at the same location within a block. (**C**) Trials in the “random location” condition: targets and their flankers were presented in any of the four locations within the block. (**D**) Trials in the “pre-cue” condition: a target was presented at a randomly chosen location, which was pre-cued.

Stimuli were generated using MATLAB (Mathworks, Natick, MA, USA) with Psychtoolbox extensions (Brainard, 1997; Kleiner, Brainard, & Pelli, 2007; Pelli, 1997) and presented on LCD monitors (53 cm width, 1920 × 1080 pixels, 60 Hz, University of Aberdeen; 47.6 cm width, 1920 × 1080 pixels, 60 Hz, University of Essex). A black square frame whose side was five pixels less than the height of the monitor was presented throughout the experiment to indicate the area within which the stimuli would be presented. Stimuli were nine black letters in the Sloan font (D, H, K, N, O, R, S, V, and Z) presented on a grey background (Figure 1). The letter C was omitted because C and O are much less discriminable than other pairs in the Sloan font (Elliott, Whitaker, & Bonette, 1990; Song, Levi, & Pelli, 2014). One target and one flanker from this set were randomly chosen on any given trial. The target was presented at 7.5 degrees eccentricity at one of four locations (left, right, above, or below the fixation, on the horizontal or vertical meridian). Pairs of white lines, of length 2 degrees each, were presented on either side of the four possible target locations to reduce confusion about which of the two letters was the target. The lines were oriented perpendicular to the meridian across which they were arranged. The gap between these lines was set to twice the letter size (which was scaled on each trial; see below). We had piloted a version where the lines were fixed at the same locations on all trials, but observers reported that this still led to confusions regarding which of the letters was the target, particularly at small letter sizes. We believe that these lines would cause no or minimal crowding themselves, as they were aligned in the tangential direction and differed in contrast polarity, size, and complexity from the target (Kooi, Toet, Tripathy, & Levi, 1994; Toet & Levi, 1992; Zhang, Zhang, Xue, Liu, & Yu, 2009).

A black fixation cross (0.3 degrees) was presented throughout the trial. To ensure fixation, eye movements were monitored using a desktop mounted Eyelink 1000 eye tracker (SR Research Ltd., Mississauga, Ontario, Canada) with a sampling rate of 1000 Hz. The right eye was tracked, and a five-point calibration was performed at the beginning of each block. The same eye tracker make and model was used at both sites.

In some conditions, a pre-cue was presented at the location of the target to draw exogenous attention to that location (Nakayama & Mackeben, 1989). The pre-cue was a red circle of diameter 0.3 degrees, presented 100 ms before target onset and lasted for 50 ms.

### Design

Participants were tested on three design conditions: blocked, random, and pre-cued. In the blocked design condition, within a given block the target was presented at the same chosen location. In the random design condition, the target was presented in any of the four locations with equal probability. Finally, in the pre-cued design condition, the target was presented in any of the four locations with equal probability, but this location was indicated with a pre-cue presented at the target location before target onset. Each design condition was tested in a separate block and the order of blocks was randomized. Each participant completed eight blocks of each design condition. These 24 blocks (3 design conditions × 8 blocks each) were tested over four sessions (six blocks per session), which were, in most cases, run on separate days. Each block included 120 trials, leading to a total of 2880 trials per participant.

The target was presented in isolation (unflanked) or along with a single flanker. When presented, the flanker would be placed either on the inward (or foveal) side of the target or on the outward (or peripheral) side of the target. All flanker locations (inward, outward, and none) were equally represented within each block. Overall, we assessed performance in three design conditions (blocked, random, and pre-cue), four target locations (up, down, left, and right) and three flanker conditions (in, out, and no flanker), for a total of 36 combinations.

### Procedure

Participants were seated 72 and 60 cm from the monitor, at the Aberdeen and Essex sites, respectively, and their heads were stabilized with a chin and forehead rest. In all conditions, each trial began with a variable fixation period of between 900 and 1500 ms in steps of 50 ms, randomly chosen. Fixation was monitored continuously with the eye-tracker. The trial was initiated only if the participant fixated within 100 pixels of the center of the screen for 1000 ms. The target was then presented for 100 ms in isolation or with a single flanker placed either inward or outward relative to the target. All nine possible letter identities were then displayed on the screen and the participant reported the identity of the target letter with a mouse click. Feedback was provided 200 ms after the response as a color change of the fixation cross: it briefly (300 ms) turned green if the response was accurate and red if it was incorrect.

Following Song, et al. (2014) and Petrov and Meleshkevich (2011a, 2011b), we compared flanked with unflanked visual acuity to assess the extent of crowding in each of the 36 condition combinations. To do so, we first obtained acuity thresholds in each of the conditions using the QUEST algorithm, an adaptive staircase that controlled the size of the letters on a trial-by-trial basis. In the case of flanked acuity, the center-to-center spacing between the target and the flanker was fixed at 1.1 times the letter size, such that the spacing scaled with size, in order to prevent overlap and to measure the extent of crowding. The letter size was constrained to be between 0.1 degrees and 3 degrees, a relatively large range of sizes. The size threshold for each condition was estimated in two QUEST runs of 40 trials each. The 80 trials of these two runs were then pooled and (re)subjected to the QUEST algorithm to obtain a single threshold per condition. The parameters used for QUEST were: chance rate = 0.11 (1 out of 9); slope of psychometric curve = 3.5; initial guess of acuity threshold = 1.1 degrees; standard deviation of the initial guess = 3; lapse rate = 0.05; and threshold criterion = 0.5.

The ratio of flanked to unflanked acuity, called the crowding factor (Petrov & Meleshkevich, 2011a, 2011b), specifies the extent of crowding. A crowding factor of greater than one indicates the presence of crowding; the larger the factor, the larger the target-flanker interference zone. We then computed the ratio of crowding factors for outward and inward flankers, which is equivalent to computing the ratio between the size threshold in the presence of the outward flanker and the size threshold in the presence of the inward flanker. A ratio of greater than one indicates IOA, where the outward flanker is more effective at crowding than the inward flanker.

### Data analysis

In general, we have followed the advice on Bayesian modeling given by McElreath (2015). We used multilevel Bayesian linear regression (Bürkner, 2018), with a log-normal distribution, to model the relationship between design (blocks/randomized/pre-cued), location (up/left/down/right), flanker (none/inward/outward), and a participant's letter identification threshold. All interactions were included, and all main effects were allowed to vary from observer to observer, allowing us to characterize in-out asymmetry and the variability of these estimates. We note that we did not log-transform the thresholds and fit the regression model to the transformed variables; instead, we assumed that the thresholds were drawn from a log-normal distribution rather than from a normal one. The log-normal distribution was used as the thresholds are bounded by zero and were expected to have a positive skew.

We used a set of informed conservative priors for our analysis, based on the following assumptions:

i) For the no-flanker condition, the thresholds for each of the four target locations were assigned N(1,2) priors (i.e. there is a 75% chance that the threshold lies between 0.05 degrees and 5 degrees), with a median of around 1.6 degrees. Here and elsewhere, N(µ, σ) represents a normal distribution with a mean of µ and a standard deviation of σ.
ii) Threshold sizes in the flanked conditions are expected to be larger (Levi, 2008; Petrov & Meleshkevich, 2011a, 2011b). Since this study is intended to specifically investigate the difference between inward and outward flankers, we conservatively assumed that both effects are equal, and used an N(1, 0.5) distribution to describe them. These conditions were dummy coded, so this N(1, 0.5) distribution is combined with the intercept, N(1, 2) to give our prior predictions for these conditions. On average, we expect the thresholds in these conditions to be between 0.1 degrees and 16 degrees with a median of around 5.0 degrees.
iii) Similarly, since we are also interested in the effect of design condition and their interactions, we assigned a prior distribution of N(0, 1) for each condition.
iv) Group-level (random) effects were all assigned a weak half-Cauchy(0, 10) distribution, while half-Cauchy(0, 1) was used for the residual variance (Gelman, 2006). These are often used as weak priors in Bayesian analyses as they rule out negative values (it is impossible to have a variance less than zero). Furthermore, they capture our belief that the variance should be low, while the heavy tails mean that larger than expected values have not been ruled out.

Before training the model on our data, we carried out prior predictions to check that the above gave sensible predictions. For example, a prior prediction of a threshold of less than 0 or more than 100 degrees of visual angle would be implausible and hence the prior would be unacceptable. The priors listed above lead to a 75% highest density interval of approximately 0.05, 15 degrees, which seems reasonable. Further details and full model specification, data and R code is available at https://osf.io/jdfmn/.

The model was fit using R (version 4.03; R Core Team, 2020) with the rStan (version 2.21.2; Carpenter, Gelman, Hoffman, Lee, Goodrich, Betancourt, Brubaker, Guo, Li, & Riddell, 2017) and brms (version 2.14.4; Bürkner, 2017) packages. Highest posterior density intervals (HPDIs) were calculated using the HDInterval package (version 0.2.2).

## Results

Thresholds estimated by QUEST with a standard deviation greater than 0.2 log units were discarded (Reuther & Chakravarthi, 2014), as these are not reliable. Sixty-one out of 1368 thresholds (38 participants times 36 thresholds) were thus discarded (4%). A summary of these data is presented in Supplementary Table S1.1 (all supplementary materials are available as an html file at https://osf.io/bcgz4/).

The multilevel lognormal model outlined in the data analysis section was successfully fit to these thresholds, with $\hat{R}$ = 1 for all parameters. The Bayesian R^2^ (Gelman, Goodrich, Gabry, & Vehtari, 2019) of the regression model, which is analogous to the standard R^2^, was 0.78 (97% HPDI = [0.77, 0.79]). We proceed to analyze the model in more detail below.

### Crowding and asymmetries in an average observer

#### Crowding

**Figure 2.**
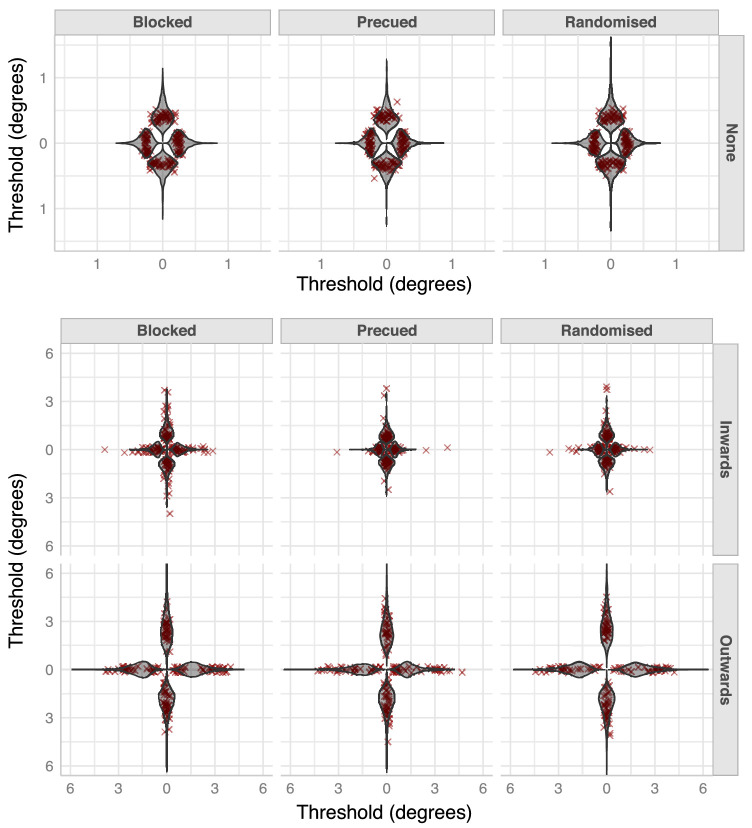
**Observed and estimated identification thresholds.** The top row represents size thresholds for unflanked letters at each of four target locations (up, down, left, and right) and three design conditions (blocked, pre-cued, and randomized). The middle and bottom rows represent size thresholds for target letters flanked by inward and outward letters, respectively. The target letters were always presented at a distance of 7.5 degrees from fixation in one of four locations. In all panels, each grey violin plot represents our model's posterior distribution of threshold size for a target letter at that location (the size of the target that allowed an identification accuracy of 50%). The red x's represent individual observers’ threshold. Note the difference in scale between the top row and the other two (middle and bottom) rows.

**Figure 3.**
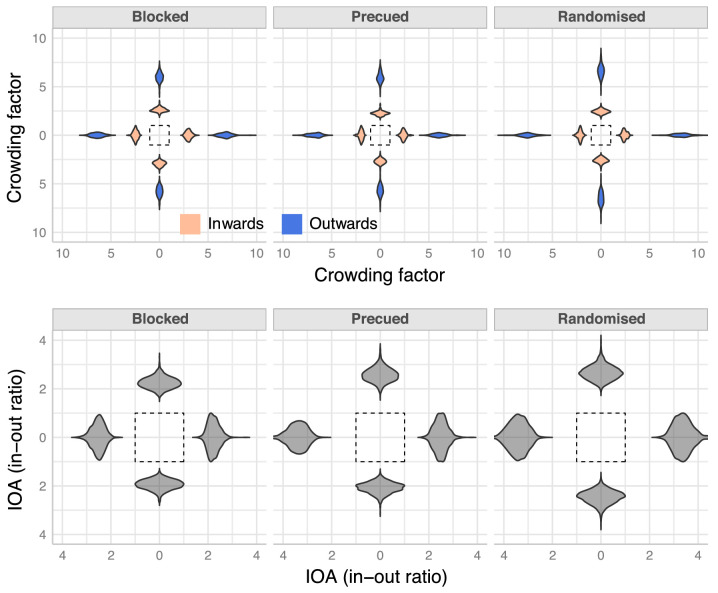
Crowding factors and in-out ratios. The top row plots the posterior distributions of crowding factors at each location and design condition for targets in the presence of either an inward (peach) or an outward (blue) flanker. Crowding factor is defined as the ratio of size thresholds of a flanked target and an unflanked target. A ratio of 1 (indicated by dashed lines at 1 in the plots) indicates no crowding. Any value greater than one indicates crowding. It is evident that an outward flanker leads to stronger crowding than an inward flanker. The violin plots in the bottom row represent the in-out ratio, which is ratio of the size threshold of a target flanked by an outward letter and that of a target flanked by an inward letter. A ratio of more than one (indicated by dashed lines at 1) indicates IOA. The IOA is evident in all locations and conditions.

We first analyzed the fixed effects of our model (i.e. the model's predictions for how the average observer would behave). Figure 2 shows that the addition of a flanker increased identification thresholds at all target locations and conditions. That is, both a single inward and a single outward flanker led to crowding. To quantify these observations, we computed the crowding factor, which is a measure of the strength of crowding (similar to the idea of threshold elevation), from the model's posterior distributions. The crowding factor is defined as the ratio between the size threshold in the presence of a flanker (either inward or outward) and the threshold in the absence of a flanker (Figure 3, top row). Crowding factors were greater than one in all design conditions, target locations, and flanker positions.

#### In-out asymmetry 

Importantly, Figure 3 (top row) shows that the crowding factors for the outward flanker (blue) were larger than the factors for the inward flanker (peach). To quantify the extent of this IOA, we computed the ratio between the size threshold in the outward flanker condition and the threshold in the inward flanker condition (see Figure 3, bottom row). Note that this is equivalent to the ratio of crowding factors for the outward and inner flankers. The plots show that the IOA was present in all conditions (*p(ioa > 1* | data*)* > 0.99) with identification thresholds between 1.5 and 4.5 times larger when an outward flanker was present compared to an inward flanker. The HPDIs are given for each condition in Table 1. There clearly are differences in the strength of IOA across target locations and design conditions. These are discussed below.

**Table 1.** Characterizing IOA. The median in-out ratio, 53%, and 97% HPDIs of the ratio in each design condition and target location are shown. A ratio of greater than one indicates IOA, where an outward flanker causes more crowding than an inward flanker. The first column indicates the probability of observing an IOA greater than 1.5 (a conservative estimate of IOA). An IOA of at least 1.5 can be reliably observed in nearly all conditions. The probability that the IOA ratio was greater than one was >0.99 in all conditions and is not shown here. The remaining columns indicate the range of the IOA ratio in our posterior predictions.

Location	Design	*p*(ioa > 1.5)	Lower end of 97% HPDI	Lower end of 53% HPDI	Median	Upper end of 53% HPDI	Upper end of 97% HPDI
Right	Blocked	>0.99	1.74	2.10	2.21	2.45	2.76
	Pre-cued	>0.99	1.96	2.24	2.50	2.66	3.10
	Random	>0.99	2.49	3.21	3.39	3.73	4.43
Up	Blocked	>0.99	1.79	2.08	2.21	2.42	2.70
	Pre-cued	>0.99	1.90	2.46	2.62	2.91	3.12
	Random	>0.99	1.95	2.41	2.67	2.82	3.33
Left	Blocked	>0.99	1.93	2.30	2.51	2.69	3.30
	Pre-cued	>0.99	2.33	3.06	3.38	3.55	4.22
	Random	>0.99	2.73	3.09	3.38	3.66	4.35
Down	Blocked	>0.99	1.55	1.74	1.97	2.07	2.39
	Pre-cued	0.99	1.57	1.88	2.09	2.24	2.54
	Random	>0.99	1.91	2.27	2.41	2.63	3.01

#### Visual field asymmetries in IOA

Recent studies on IOA presented inconsistent results in relation to its distribution across the visual field. It was argued that it was chiefly observed along the horizontal meridian but not at other locations (Petrov & Meleshkevich, 2011b). To characterize the pattern of IOA across the visual field, we analyzed the effect of target location. First, we computed the ratios between the IOA at the up target location and the IOA in the down location. As we can do this repeatedly using different samples from the model's posterior probability distribution, we can easily compute the various conditional probabilities (Supplementary Table S4.2). IOA was stronger (more extreme) in the upper visual field compared to the lower visual field. This effect appears to be particularly robust in the blocked and pre-cued designs (both *p* = 0.98), and much weaker (approximately *p* = 0.7) in the randomized condition. Similarly, IOA was stronger in the left visual field compared to the right, with a similar influence of design. Finally, we pooled the IOA ratios for locations along the horizontal meridian (left and right) and compared these to IOA ratios pooled along the vertical meridian (up and down). IOA was stronger along the horizontal meridian (left and right) compared to the vertical with *p* ≥ 0.99 across all three experimental designs. In summary, we found a vertical asymmetry, a horizontal asymmetry and a horizontal-vertical asymmetry in IOA. However, as noted above, IOA was present at all locations.

#### The effect of attention and location uncertainty on IOA

**Figure 4.**
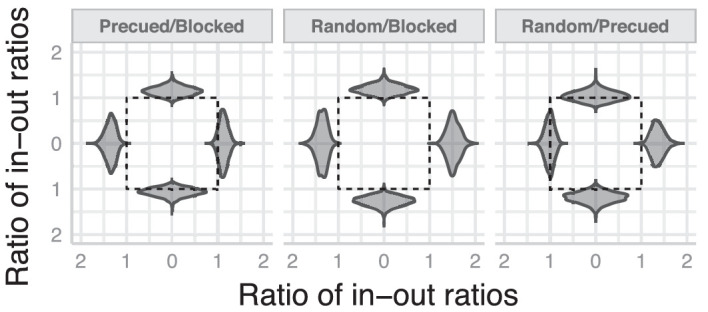
**The effect of design condition on IOA.** The ratio of in-out ratios between different design conditions are depicted as violin plots. A ratio of one (dashed lines) marks no difference between design conditions.

One of the motivations of the current study was to assess the effect of target location uncertainty on IOA. Previous studies had suggested that if targets were presented at a single location, IOA would be greatly diminished or disappear, whereas if the location was uncertain, IOA would be manifest, particularly along the horizontal meridian (Petrov & Meleshkevich, 2011b). Further, modulating attentional allocation affected and sometimes even reversed the IOA (Petrov & Meleshkevich, 2011a). To test these observations, we assessed the differences in IOA across experimental designs (blocked, pre-cued, and randomized). Figure 3 (bottom row) suggests that IOA was weaker in the blocked condition than in the randomized condition. This difference is more evident at horizontal locations compared to vertical ones. To quantify these observations, we computed pairwise ratios between IOA in the pre-cued, blocked, and randomized conditions at each target location (Figure 4). When target locations were known for the entire block (blocked design), and hence voluntary attention could be allocated in advance, IOA was weaker than when attention was directed to the target location exogenously just before the target was presented (pre-cued). That is, reducing location uncertainty by diverting attention to the target location was not sufficient to render IOA comparable to when there was no location uncertainty. Exogenous attention can mitigate but not eliminate the asymmetry. Interestingly, pre-cueing the location of the target had a nuanced effect on the strength of asymmetry compared to when location uncertainty was maximum (random target location). It reduced IOA at some locations (right and down) but not at others (left and up). Finally, IOA in the randomized target location condition was much stronger than in the blocked condition. That is, sustained attention to one location mitigated the IOA present when target location was uncertain. In summary, IOA was strongest under conditions of target location uncertainty. Pre-cueing the target location and thus reducing target location uncertainty partially reduced the asymmetry. However, advance knowledge of the target's location substantially reduced IOA. Note that, importantly, IOA was not eliminated in any condition or location; at all locations, the outward flanker was at least two times more effective at interfering with the target than the inward flanker.

### Individual differences in IOA

The fixed effects reported above give compelling evidence that there is IOA in the “average participant.” However, as we can see from Figure 2 (red x's) there is substantial variation in the identification thresholds across participants. To gain further insight into individual differences, using our model, we simulated a set of 1000 participants and calculated the proportion of this sample that exhibits the IOA effects discussed above (Supplementary Table S4.3). We found that a large majority of the new sample displayed IOA in all locations and design conditions, suggesting that even when taking into account the fact that there are considerable individual differences, the presence of IOA is reliable. The median and range of IOA in this reconstructed sample was wider than that for the “average participant” (by “average,” we mean the fixed effect estimates discussed above, and not the raw empirical means). More importantly for our main question of whether IOA can be considered a diagnostic marker of crowding, there was a small minority of the sample in whom the IOA was not observed. This was the case if we used a conservative criterion for establishing the asymmetry (that is, whether the ratio of the effect of the outward flanker was 1.5 times that of the inward flanker). A less stringent criterion (ratio > 1) indicated that IOA was observable in almost all participants at all locations and conditions. The effect of target location and design appears to be the same as for the average participant.

## Discussion

The IOA has been argued to be a diagnostic marker of crowding, superior to other previously considered criteria of crowding, such as scaling with eccentricity in the absence of scaling with size (Petrov et al., 2007). However, there have been reports of inconsistencies in its strength and prevalence, both among individuals and across visual field locations (Petrov & Meleshkevich, 2011a, 2011b; Strasburger, 2020). Hence, we undertook a comprehensive study of IOA in a large cohort of participants at a range of visual field locations under multiple conditions. Our findings indicate that IOA is present in the vast majority of observers, locations, and conditions. It is definitively present at all locations under diverse conditions of target uncertainty and attentional allocation when computed as an average across a population sample. However, there are considerable individual differences, with a small minority of individual participants not displaying or weakly displaying the asymmetry, particularly at some locations (e.g. lower visual field). These latter observations, however, are based on a conservative criterion applied to the estimated IOA as a measure of its presence (the proportion of a simulated population in whom the outward flanker's crowding effect is at least 1.5 times that of the inward flanker's crowding effect). A more relaxed criterion (IOA ratio > 1) shows that IOA is present at all locations under most conditions in the vast majority of individuals. Hence, IOA can serve as a diagnostic marker of crowding across a sampled population and also among individuals, provided that this asymmetry is not observed in other flanker-induced interference phenomena, such as masking (Petrov et al., 2007).

Our results indicate that IOA is modulated by attentional allocation. IOA was strongest when there was substantial target location uncertainty (target location randomly picked from among four possible options on each trial). However, in such a situation, if the target location was indicated by a brief pre-cue, IOA was reduced at some, but not all, locations. That is, exogenous attention drawn to the target location, under conditions of location uncertainty, mitigates the IOA at least in some locations. More interestingly, in situations where there was no target location uncertainty, and hence when sustained attention could be allocated to the location, the IOA was the weakest (albeit still observable). These findings suggest that the type and mode of attentional allocation modulates the strength of asymmetry. Previous accounts have also found that attentional allocation modulates IOA, which have led to the proposal that the occurrence of IOA can be attributed to asymmetrical deployment of attention (Petrov & Meleshkevich, 2011a). Our findings are in line with this possibility but are also compatible with the proposal that there is biased sampling of the outward flanker (Shechter & Yashar, 2021). The latter argues that crowding arises due to pooling of features where a weighted sum of available features is taken within a receptive field. IOA arises in these circumstances because the immediately outward flanker is assigned a higher weight, leading to more frequent misreports of this flanker relative to other objects. Although these two mechanisms implicitly point to different underlying mechanisms, in practical terms, they are hard to distinguish. An attentional bias toward the outer flanker can be argued to be equivalent to higher weights assigned to it. Further tests would be needed to disentangle the two. One issue with the disproportionate weighting hypothesis is that it is not clear how this would apply to complex objects, including letters. A weighted sum model can well explain crowding and IOA for simple features, such as orientation and motion (Harrison & Bex, 2015; Shechter & Yashar, 2021; van den Berg, Roerdink, & Cornelissen, 2010), but the details for applying such a model to the relevant features of complex objects need to be worked out, although there have been impressive steps along this direction (Keshvari & Rosenholtz, 2016).

As noted above, IOA was observed at all tested locations, even when there was no location uncertainty of the target. Few studies have tested IOA at locations other than along the horizontal meridian. Petrov and Meleshkevich (2011a, 2011b) reported that IOA was observed only along the horizontal meridian and only if multiple locations were monitored. The discrepancy between their results and ours can probably be attributed to a couple of factors: (a) they used coarse discrimination tasks (2AFC) that might not have been sensitive enough to detect the slightly weaker IOA along the vertical meridian (and presumably other locations), and (b) sampling: we found that a minority of our participants do not exhibit IOA at some locations; it could be that previous studies might have inadvertently tested a small sample of participants with minimal or no IOA at some locations.

The strength of IOA is not the same across the visual field; that is, we observed a range of visual field asymmetries in IOA. The IOA is stronger in the upper visual field than in the lower visual field (vertical asymmetry [VA]); in the left visual field than in the right (horizontal asymmetry [HA]); and along the horizontal meridian than along the vertical meridian (horizontal vertical asymmetry [HVA]). These asymmetries are reminiscent of visual field asymmetries observed across many visual tasks (Carrasco, Talgar, & Cameron, 2001) including crowding. Crowding is known to be worse in the upper than in the lower visual field (VA; Intriligator & Cavanagh, 2001) and worse along the vertical meridian than along the horizontal meridian (HVA; Greenwood et al., 2017). Crowding is also worse in the left visual field than in the right visual field (HA; Greenwood et al., 2017; Kurzawski, Burchall, Thapa, Majaj, Winawer, & Pelli, 2021). At first glance, it might appear that the asymmetries in IOA are simply inherited from already prevalent asymmetries in crowding. However, it is clear from the above that, whereas there is some alignment in asymmetries, there are notable differences. Both IOA and crowding are stronger in the upper visual field compared to the lower visual field (VA). Similarly, both IOA and crowding are stronger in the left visual field than in the right (HA). However, IOA is stronger while crowding is weaker along the horizontal meridian than along the vertical (HVA). Further, the strongest asymmetry observed in crowding is the HVA (Kurzawski et al., 2021), which is the one that shows the reversal in asymmetry for IOA. This suggests that asymmetries in IOA are not simply inherited from crowding asymmetries. Nevertheless, it would be useful to keep in mind the admittedly more complex situation where the two asymmetries that do align are inherited, but the discrepant asymmetry (HVA) is affected by additional causes. This is not a parsimonious explanation, but our data are consistent with this possibility and hence it cannot be ruled out.

Some of the asymmetries in crowding have previously been explained in terms of attentional allocation, which might be taken to suggest that asymmetries in IOA can be attributed to asymmetries in attentional allocation. The resolution of attention is better in the lower visual field than in the upper visual field (Intriligator & Cavanagh, 2001). Performance in tasks that rely on sustained attention is also thought to be better along the horizontal meridian than along the vertical meridian (Mackeben, 1999). Further, whereas selective attention appears to work equally well in both left and right visual fields (Arguin, Joanette, & Cavanagh, 1990; Corbetta, Miezin, Shulman, & Petersen, 1993; Yamaguchi, Tsuchiya, & Kobayashi, 1994), there is evidence that attention selects and processes stimuli better in the left visual field than in the right visual field (Asanowicz, Śmigasiewicz, & Verleger, 2013; Evert, McGlinchey-Berroth, Verfaellie, & Milberg, 2003; Goodbourn & Holcombe, 2015; Matthews & Welch, 2015; Newman, Loughnane, Kelly, O'Connell, & Bellgrove, 2017; Verleger, Śmigasiewicz, & Möller, 2011). However, as with crowding asymmetries, the relationship between asymmetries in attentional tasks and those in IOA is inconsistent. Performance in attentional tasks is better along the horizontal meridian than along the vertical. Correspondingly, IOA is more extreme along the horizontal meridian than along the vertical. Further performance is often better in the left visual field than in the right, and IOA is stronger in the left visual field. In these cases, the strength of IOA and attentional allocation seem to be positively correlated. In contrast, although the resolution of attention is better in the lower visual field relative to the upper visual field, IOA is less extreme in the lower visual field. Here, the strength of IOA seems to be inversely correlated with attentional allocation at these locations. That is, there does not seem to be a clear relationship between performance in attentional tasks and the strength of IOA, suggesting that attention cannot be the whole story. A nuance to this conclusion is the finding that when monitoring two rapid serial visual presentation streams, attention is better at selecting a target in the upper visual field than the one in the lower visual field target just as it preferentially selects a target in the left stream relative to the one on the right (Goodbourn & Holcombe, 2015). This indicates better attentional selection in the left and upper visual fields compared to the right and lower visual fields, which might potentially explain the stronger IOA in these locations. This would account for all the observed asymmetries in IOA. However, such asymmetrical attentional processing was observed only when two locations were simultaneously monitored and was absent when a single stream was attended. In our experiment, we found the same pattern of IOA asymmetries irrespective of whether multiple locations had to be monitored (random design block) or when a single location was to be monitored for targets throughout the block (blocked design condition and arguably the pre-cued design condition). Hence, the preferential attentional processing observed only when multiple simultaneous targets were monitored and reported might not help elucidate the mechanisms underlying the asymmetries observed in this study.

A speculative proposal to explain asymmetries in IOA is that the stronger asymmetry observed in the left visual field and also along the horizontal meridian might be due to the ease of processing letters at these locations acquired through years of reading experience (in languages that are read left to right, as was the case for the participants tested in this study). The participants might assign higher weights to the outermost letter(s) on the left side while reading, which leads to stronger IOA in the left visual field and a weaker one in the right visual field. The same reasoning can help explain the stronger IOA along the horizontal meridian relative to the vertical meridian. Because reading experience does not lead to higher weights to letters along the vertical meridian, the IOA along that axis tends to be weaker. Notwithstanding these possibilities, this “reading experience” hypothesis does not explain the observed asymmetry between upper and lower visual fields (without some contortions).

## Conclusion

We conducted an extensive examination of the IOA in crowding in a relatively large sample of observers. The study was designed to characterize IOA at multiple visual field locations under various conditions of location uncertainty and attentional allocation. We found that the IOA is observable in all tested locations and conditions in the vast majority of participants. We conclude that it is a reasonable candidate to diagnose crowding to the extent that it can distinguish crowding from various other flanker-induced interference phenomena such as lateral masking, while keeping in mind that a small minority might not display the asymmetry at some locations.

## Footnotes

1 This study is part of a pre-registered project (the first experiment at this link: https://osf.io/jdfmn/).

2 We briefly note the sample sizes used in selected previous studies. Petrov et al., 2007: 5; Petrov & Meleshkevich, 2011a, 2011b: 6; Shechter and Yashar, 2021: 13–14; Strasburger and Malania, 2013: 9–10.
